# Believing in food addiction: Helpful or counterproductive for eating behavior?

**DOI:** 10.1002/oby.21499

**Published:** 2016-05-05

**Authors:** Helen K. Ruddock, Paul Christiansen, Andrew Jones, Eric Robinson, Matt Field, Charlotte A. Hardman

**Affiliations:** ^1^ Department of Psychological SciencesUniversity of LiverpoolUK; ^2^ UK Centre for Tobacco and Alcohol StudiesUK.

## Abstract

**Objective:**

Obesity is often attributed to an addiction to food, and many people believe themselves to be “food addicts.” However, little is known about how such beliefs may affect dietary control and weight management. The current research examined the impact of experimentally manipulating participants' personal food addiction beliefs on eating behavior.

**Methods:**

In two studies, female participants (study 1: *N* = 64; study 2: *N* = 90) completed food‐related computerized tasks and were given bogus feedback on their performance which indicated that they had high, low, or average food addiction tendencies. Food intake was then assessed in an *ad libitum* taste test. Dietary concern and time taken to complete the taste test were recorded in study 2.

**Results:**

In study 1, participants in the high‐addiction condition consumed fewer calories than those in the low‐addiction condition, *F*_(1,60)_ = 7.61, *P* = 0.008, *η*_p_^2^ = 0.11. Study 2 replicated and extended this finding, showing that the effect of the high‐addiction condition on food intake was mediated by increased dietary concern, which reduced the amount of time participants willingly spent exposed to the foods during the taste test, *b* = −0.06 (0.03), 95% confidence interval = −0.13 to −0.01.

**Conclusions:**

Believing oneself to be a food addict is associated with short‐term dietary restriction. The longer‐term effects on weight management now warrant attention.

## Introduction

Obesity continues to increase, with more than half of adults worldwide now having overweight or obesity 1. Overeating and obesity are frequently attributed to a food‐based addiction though this notion has been the source of considerable controversy within the scientific community 2, 3, 4. However, scientific understanding has not kept pace with the lay public's enthusiasm for the concept of “food addiction” 5, 6, 7, 8. Indeed, in a recent study, almost three quarters of participants believed that obesity is caused by an addiction to certain foods 7. Furthermore, as many as 50% of people believe themselves to be food addicts 9, 10. To date, little is known about the potential impact of believing oneself to be a food addict on eating behavior.

An addiction‐based explanation implies that excessive eating is outside of personal control and, in this way, may help to remove individual responsibility for overconsumption 11. However, there may be counterproductive effects on eating behavior. It is well‐established that feeling in control of one's behavior is important for health and predicts engagement in a variety of health‐promoting dietary behaviors 12, 13, 14. Conversely, public health messages which imply a lack of personal control over behavior (e.g., “obesity is a disease”) have been associated with unhealthy food choices and greater food intake 15, 16.

An opposing idea is that food addiction may be *helpful* for the initiation of healthy dietary behaviors. Notably, members of Overeaters Anonymous reported an increased sense of responsibility after acknowledging their “addiction” to food 17, 18. Furthermore, diminished self‐control beliefs may lead people to avoid putting themselves in tempting situations in the first place. In one study, smokers who were told that they had a low capacity for self‐control subsequently exposed themselves to fewer tempting smoking scenarios, and were thus less likely to smoke, than participants who were told they had a high capacity for self‐control 19. Consistently, participants who were told that they had low self‐control consumed less alcohol in a subsequent “taste test” than those who believed that they had high self‐control 20.

To test these possibilities, the current research aimed to experimentally manipulate participants' personal beliefs about food addiction—that is, the extent to which they believed themselves to be food addicts. In a previous study, beliefs about the existence of food addiction (e.g., “food addiction is real”) were found to be malleable though there was no clear effect of this belief manipulation on food consumption 9. However, in this study, we reasoned that leading people to believe that they are personally affected by food addiction would be more likely to influence subsequent eating behavior. This is supported by evidence that personalized feedback is highly effective at invoking dietary behavior change 21. In a two‐tailed hypothesis, we predicted that believing oneself to be a food addict would *either* promote overconsumption due to reduced personal responsibility for eating *or* cause a person to be concerned about their eating behavior and consume less snack food.

## Study 1 Methods

### Participants and design

Female university staff and students (*N* = 64) were recruited to take part in a study into the effect of mood on taste preferences. As this study was the first to examine the effect of personal food addiction beliefs on food intake, we restricted the sample to female participants in order to minimize differences between‐subjects. Indeed, gender differences in dietary beliefs and the prioritization of healthy eating behaviors have previously been demonstrated 22. In a between‐subjects design, participants were randomly allocated to either high‐addiction or low‐addiction conditions. As there were no previous studies upon which to base an *a priori* power analysis, we selected a target sample size of 60, with 30 participants per cell, which would give 86% power to detect a large effect (*f* = 0.4) at an *α* level of *P* = 0.05 (GPOWER 3.1 23). We slightly over‐recruited to account for any participants guessing the aims of the study. Participants were asked not to eat or consume any calorie‐containing drinks for 3 h before the study and all indicated compliance with this instruction. Ethical approval was granted by the University Research Ethics Committee and all participants gave written informed consent before participation.

### Measures and procedure

We adapted the methodology used by Jones et al. 20 in order to manipulate personal food addiction beliefs. Participants first completed modified, food‐related versions of the valence implicit association task (IAT) 24, 25 and a standard stop‐signal task (SST) 26, which they were told would assess their addictive tendencies towards food. Following each task, a bogus score was displayed on the computer screen. The experimenter explained that the score reflected either a high (in the high‐addiction condition) or low (in the low‐addiction condition) tendency towards food addiction. To further enforce believability, participants were shown a bogus histogram which ostensibly illustrated the distribution of food addiction scores within the general population. Those in the low‐addiction condition were informed that they had scored within the bottom‐quartile of the distribution, while those in the high‐addiction condition were told that they scored within the top‐quartile. Participants then completed one of two versions of a leading questionnaire to reinforce beliefs about their own level of food addiction. In the high‐addiction condition, the questionnaire consisted of five items that were congruent with addictive‐like eating behavior which participants would be likely to agree with (e.g., “I sometimes crave sweet, salty, or fatty foods”). In the low‐addiction condition, the questionnaire consisted of five items that were congruent with non‐addictive‐like eating (e.g., “I usually feel in control of what and how much I eat”). In response to each question, participants could tick either “yes” or “no.”

Participants were then asked “Do you believe that some people are addicted to food?”, to which they could tick either “yes” or “no.” This question was included to ensure that all participants believed in the concept of food addiction and thus were potentially susceptible to the manipulation. Next, to ensure that the manipulation had been successful, participants completed a measure of self‐perceived food addiction (i.e., “I believe myself to be a food addict”). Responses were provided on a 5‐point scale from strongly disagree to strongly agree. They then rated their levels of hunger and fullness, and completed a set of 21 mood ratings (e.g., nervous, miserable; these were included to provide consistency with the cover story that the study was exploring the effects of mood on taste). A complete list of mood ratings is provided in the Supporting Information. All ratings used 100 mm visual analogue scales (VAS) that were anchored by “not at all” on the left and “extremely” on the right. Next, participants completed the taste task in which they were provided with a 50 g bowl of crisps (Tesco Ready salted crisps: 454 kcal/100 g, 33.2 g fat/100 g) and a 100 g bowl of chocolate (Cadbury Dairy Milk Giant Buttons: 530 kcal/100 g, 30 g fat/100 g). Before tasting each food, participants completed 100 mm VAS ratings of “expected liking,” “desire to eat,” “craving,” and “difficulty to resist.” Participants were then instructed to consume as much of the food as they wished, and to rate each food on seven taste scales (e.g., salty, sweet). A complete list of taste ratings is provided in the Supporting Information. Participants ended the taste task whenever they wished. Following the taste task, participants rated their hunger and fullness again, as well as how much they enjoyed eating each food, using 100 mm VAS.

To monitor demand characteristics, participants were asked to write down what they believed to be the aims of the study. They then completed the Yale Food Addiction Scale (YFAS) 27, and the restraint and disinhibition subscales of the Three‐Factor Eating Questionnaire (TFEQ‐R and TFEQ‐D, respectively) 28. The YFAS uses the diagnostic statistical manual (DSM‐IV) criteria for substance dependence to measure and diagnose dependence on high‐fat and high‐sugar foods. The TFEQ‐R and TFEQ‐D assess individual tendencies to restrict food intake, and to overeat, respectively. Finally, measures of height (in m) and weight (in kg) were taken and used to calculate body mass index (BMI). At the end of the study, participants were fully debriefed and informed that the food addiction feedback was bogus.

## Study 1 Results

Two participants did not believe in the concept of food addiction, one of whom also guessed the aims of the study. These two participants were removed from subsequent analyses^1^. The remaining sample consisted of *n* = 30 in the low‐addiction condition, and *n* = 32 in the high‐addiction condition. Independent samples *t*‐tests conducted on the remaining sample did not uncover any between‐condition differences with regard to BMI, age, or performance on the computerized tasks (*P*s > 0.262).

Participants in the high‐addiction condition believed more strongly that they were food addicts (*M* = 3.84 ± 0.69)^2^ than those in the low‐addiction condition (*M* = 2.77 ± 0.88), *t*_(60)_ = 5.29, *P* < 0.001, *d* = 1.35, thus indicating that the manipulation was successful.

**Figure 1:**
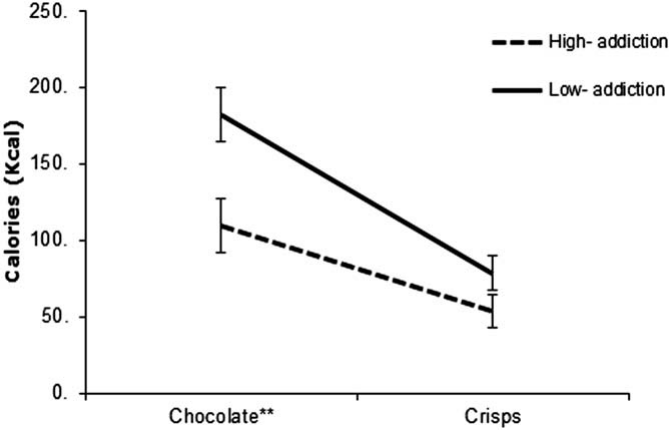
Mean calories consumed from chocolate and crisps as a function of condition. **Significant between‐condition difference at *P* < 0.01. Error bars represent standard error of the mean.

A 2 × 2 mixed design ANOVA was conducted with food (chocolate, crisps) as the within‐subjects factor, and condition (high addiction and low addiction) as the between‐subjects factor. There was a main effect of condition on calorie intake, *F*_(1,60)_ = 7.61, *P* = 0.008, *η*_p_ 2 = 0.11, such that those in the high‐addiction condition (*M* = 163.20 ± 129.47) consumed significantly fewer calories than those in the low‐addiction condition (*M* = 260.50 ± 147.62). There was also a significant condition by food interaction, *F*_(1,60)_ = 4.52, *P* = 0.038, *η*_p_ 2 = 0.07, see Figure 1. Subsequent independent *t*‐tests revealed that those in the high‐addiction condition consumed fewer calories from chocolate than those in the low‐addiction condition, *t*_(54)_ = −2.88, *P* = 0.006, *d* = 0.73. Participants also tended to eat fewer calories from crisps in the high‐addiction condition relative to the low‐addiction condition though this difference was not statistically significant, *t*_(60)_ = −1.61, *P* = 0.113, *d* = 0.41.

There were no differences between conditions with regards to hunger ratings obtained before the taste task (high‐addiction condition: *M* = 63.26 ± 20.91 mm; low‐addiction condition: *M* = 60.13 ± 20.86 mm), *t*_(60)_ = 0.590, *P* = 0.558, *d* = 0.15. Similarly, there were no between condition differences on appetite, mood, and taste ratings (see Supporting Information). Finally, YFAS symptom count^3^, and scores on the TFEQ‐R and TFEQ‐D did not differ between conditions (*Ps* > 0.352), indicating that these measures were unaffected by the bogus feedback. No participants fulfilled the YFAS diagnostic criteria for food addiction.

## Interim Discussion

Participants who were led to believe that they had scored highly on an ostensible measure of food addiction consumed less snack food than those who were led to believe that they had a low score. This is consistent with the notion that believing in food addiction may help people to limit their food intake. However, it is not possible to determine the direction of the results; calorie intake may have decreased in the high‐addiction condition, increased in the low‐addiction condition, or both. Accordingly, study 2 included a control condition in which participants were led to believe that they had “average” food addiction tendencies. Study 2 also included a direct test of the hypothesis that believing oneself to be a food addict would decrease eating because it generates concern about one's eating behavior. Specifically, it was predicted that those in the high‐addiction condition would demonstrate higher levels of dietary concern than those in the low‐addiction condition, and that this in turn would lead them to reduce the amount of time that they exposed themselves to the snack foods in the taste test. Finally, study 2 examined whether the food addiction manipulation influenced participants' more general beliefs about food‐related self‐control and their future intentions to diet.

## Study 2 Methods

### Participants and design

Ninety female participants were recruited using the same procedure as in study 1. In a between‐subjects design, participants were randomly allocated to high‐, low‐, or average‐addiction conditions. We powered the study (80% power) using GPOWER 3.1 to detect a medium‐large effect size (*f* = 0.35, on the basis of study 1) at an *α* level of *P* = 0.05 and recruited slightly above the required sample (*N* = 84) to account for any participants guessing the study aims.

### Measures and procedure

As in study 1, participants completed the computerized SST and IAT tasks and received bogus feedback about their food addiction tendencies. Those in the high‐ and low‐addiction conditions received the same feedback, and completed the same leading questionnaires as in study 1. Participants in the average‐addiction condition were provided with bogus scores following each task which they were led to believe represented average food addiction tendencies. This was further enforced by the bogus histogram on which their scores corresponded to the 50th percentile. Furthermore, those in the average‐addiction condition completed a version of the leading questionnaire that consisted of two questions from the high‐addiction condition, and two questions from the low‐addiction condition. In the interest of maintaining consistency between conditions, those in the high‐ and low‐addiction conditions completed *four* leading questions, rather than the *five* used in study 1.

The procedure was then identical to study 1, but with the following additions: firstly, following the manipulation check, participants were asked to indicate the extent to which they felt able to control their food intake. Responses were provided on an 8‐point scale ranging from “extremely poor” to “extremely good.” Secondly, the amount of time (in seconds) that participants took to complete the *ad libitum* taste test was covertly recorded by the experimenter (consistent with study 1, participants ended the taste task whenever they wished). Thirdly, after completing the *ad libitum* taste task and subsequent hunger, fullness, and enjoyment rating scales, participants indicated their level of dietary concern. Responses were provided on a 100 mm VAS which ranged from “not at all concerned” to “extremely concerned.” Finally, before completing the TFEQ‐D, TFEQ‐R, and YFAS, participants completed the Dieting Intention Scale 30.

## Study 2 Results

Participants who guessed the aims of the study (*n* = 2), or who did not believe in the concept of food addiction (*n* = 3), were excluded from analyses^4^. The remaining sample consisted of *n* = 28 in the high‐addiction condition, *n* = 29 in the low‐addiction condition, and *n* = 28 in the average‐addiction condition. One‐way ANOVAs revealed no differences between conditions with regard to performance on the computerized IAT and SST tasks, age, or BMI (*P*s > 0.106).

A univariate ANOVA revealed a main effect of condition on self‐perceived food addiction, *F*_(2,82)_ = 7.33, *P* = 0.001, *η*_p_^2^ = 0.15. Specifically, those in the low‐addiction condition believed less strongly that they were food addicts (*M* = 2.10 ± 0.72) compared with those in the high‐addiction condition (*M* = 3.00 ± 1.05), *P* < 0.001, *d =* 1.00, and the average‐addiction condition (*M* = 2.64 ± 0.87), *P* = 0.025, *d* = 0.68. Self‐perceived food addiction did not differ significantly between the high‐ and average‐addiction conditions, *P* = 0.138, *d* = 0.37.

**Figure 2:**
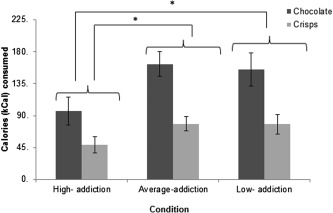
Mean calories consumed as a function of condition (high, low, or average addiction) and food type (chocolate and crisps). *Significant at *P* < 0.05. Error bars represent standard error of the mean.

A 2 × 3 mixed ANOVA was conducted with food (crisps, chocolate) as a within‐subjects factor, and condition (high, average, low addiction) as a between‐subjects factor. There was a main effect of condition on calorie intake, *F*_(2,82)_ = 3.82, *P* = 0.026, *η*_p_^2^ = 0.09 (see Figure 2). Specifically, those in the high‐addiction condition consumed significantly fewer total calories than those in the low‐, *P* = 0.024, *d* = 0.58, and average‐addiction conditions, *P* = 0.015, *d* = 0.81. Total calorie intake did not differ significantly between those in the low‐ and average‐addiction conditions, *P* = 0.837, *d =* 0.05. The condition × food type interaction was not significant, *F*_(2,82)_ = 1.30, *P* = 0.278, *η*_p_ 2 = 0.031.

Univariate ANOVAs revealed main effects of condition on dietary concern, *F*_(2,82)_ = 27.18, *P* < 0.001, *η*_p_^2^ = 0.40, and time taken to complete the taste task *F*_(2,82)_ = 5.23, *P* = 0.007, *η*_p_^2^ = 0.11. With regard to dietary concern, those in the high‐addiction condition had significantly greater levels of concern (*M* = 53.61 ± 29.68 mm) than those in the average‐addiction condition (*M* = 24.25 ± 22.66 mm; *P* < 0.001, *d* = 1.11) and low‐addiction condition (*M* = 10.17 ± 12.50 mm; *P* < 0.001, *d* = 1.91). Those in the average‐addiction condition demonstrated significantly more concern than those in the low‐addiction condition, *P* = 0.021, *d =* 0.77. With regard to time‐taken, those in the high‐addiction condition took less time to complete the taste test (*M* = 243.04 ± 109.72 s) than those in the low‐addiction condition (*M* = 369.90 ± 199.80 s; *P* = 0.007, *d* = 0.79) and average‐addiction condition (*M* = 373.32 ± 192.12 s; *P* = 0.006, *d* = 0.83). Time taken to complete the taste test did not differ between those in the low‐ and average‐addiction conditions, *P* = 0.940, *d* = 0.02.

**Figure 3:**
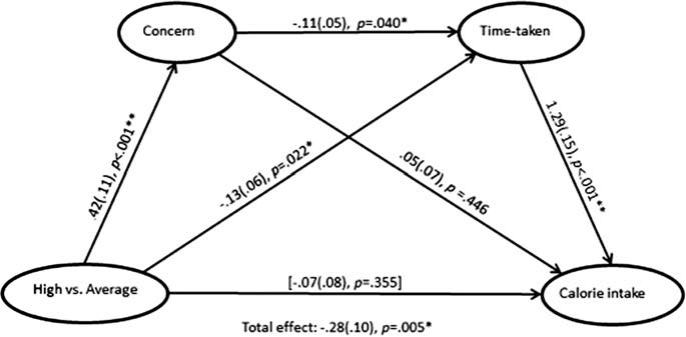
Serial mediation analysis with high versus average condition comparison as the predictor variable, calorie intake as the outcome variable, and eating behavior concern and time‐taken as first and second mediators, respectively. Values are unstandardized regression coefficients (SEs) and associated *P* values. *Significant at *P* < 0.05, **Significant at *P* < 0.001. Bracketed association = direct effect after controlling for dietary concern and time‐taken.

We predicted that the effect of condition on calorie intake would be mediated by levels of dietary concern which, in turn, would affect the amount of time participants willingly spent exposed to the food during the taste test. To test this hypothesis, a serial multiple mediation analysis was conducted using PROCESS (Model 6) 31, 32. As we had three experimental conditions, conditions were dummy coded using the average‐addiction condition as the reference category against which high‐ and low‐addiction conditions were compared (see Supporting Information). There was a significant total effect (Figure 3), and total indirect effect (Supporting Information Table S1), of the high‐ versus average‐addiction condition on calorie intake. The high‐ versus average‐addiction condition affected calorie intake serially through dietary concern and time‐taken, *b* = −0.06 (0.03), 95% confidence interval = −0.13 to −0.01. Specifically, the reduced calorie intake observed in the high‐, relative to average‐, addiction condition, was due to increased levels of dietary concern, which were subsequently associated with reduced time taken to complete the taste task. There was also a simple indirect effect of the high‐ versus average‐addiction condition on calorie intake through time‐taken, *b* = −0.17 (0.08), 95% confidence interval = −0.33 to −0.02. After controlling for these indirect effects, the direct effect of the high‐ versus average‐addiction condition on calorie intake was no longer significant (Figure 3). There was no total effect, *b =* −0.12(0.09), *P* = 0.216, or total indirect effect of the low‐ versus average‐addiction condition on calorie intake, and none of the direct or indirect pathways in this model were significant (Supporting Information Table S1).

There was no main effect of condition on self‐control ratings or Dieting Intention Scale scores (see Supporting Information). Furthermore, there were no differences between conditions with regards to hunger ratings obtained before the taste task (high‐addiction: *M* = 58.75 mm ± 16.99 mm; average‐addiction: *M* = 68.07 mm ± 15.42 mm; low‐addiction: *M* = 60.62 mm ± 23.76 mm) (*P*s > 0.161). Notably, hunger ratings were similar across studies 1 and 2. Finally, scores on the TFEQ‐R and TFEQ‐D, and YFAS symptom count^5^ did not differ between conditions (*P*s > 0.264). A *χ* ^2^ analysis confirmed that the number of people who fulfilled the YFAS diagnostic criteria for food addiction (*n* = 7) did not differ between conditions, *χ* ^2^ 2 = 1.42, *P* = 0.536.

## General Discussion

In study 1, participants who were led to believe that they scored high in food addiction consumed fewer calories than participants who were led to believe that they had scored low. Study 2 replicated and extended this finding by showing that the effect of the high‐addiction condition on food intake was mediated by increased levels of dietary concern, which subsequently reduced the amount of time participants spent tasting and consuming the foods during the taste test.

Hoyt et al. 16 recently demonstrated that an “obesity is a disease” message was associated with reduced concern for body weight and higher‐calorie food choice in individuals who have obesity. This public health message would appear to have similar connotations to the food addiction perspective in that both imply diminished personal control over eating behavior and weight status. However, contrastingly, we found that leading people to believe themselves to be food addicts *increased* concern about eating and, in turn, *reduced* food intake. This may reflect differences between giving people general versus personalized information about health 21. There may also be different underlying conceptualizations of food addiction and disease. The notion that obesity is a disease implies that it is a physiological inevitability and therefore beyond personal control 33. In contrast, in a recent survey, food addiction was regarded to be more a matter of personal choice, and less of a disease, than other addictions such as alcoholism 34. Self‐reported food addicts may therefore retain some sense of control over their “addiction.” Indeed, in this study, participants' perceptions of their ability to control food intake were not significantly influenced by the food addiction feedback. As such, believing oneself to be a food addict may not evoke the same deleterious effects on self‐regulation as holding disease‐based beliefs about one's weight.

The inclusion of a control group in study 2 clarified that food intake *decreased* in the high‐addiction condition and did not *increase* in the low‐addiction condition. Such findings offer an alternative explanation to that provided by Nordgren et al.'s 19 restraint bias theory, in which it is proposed that an overconfidence in one's ability for self‐control may cause people to overexpose themselves to tempting situations. Specifically, our findings showed that leading people to believe that they are food addicts reduced exposure to and intake of snack foods, as opposed to a counterproductive effect of overconfidence in the group who were told they scored low in food addiction.

Few participants correctly guessed the aims of the research, suggesting that the food addiction feedback was a plausible manipulation. However, a potential limitation is that our findings may have been driven by participants' desire to prove the experimenter wrong. Specifically, upon receiving feedback that they had scored highly in food addiction, participants may have refrained from eating large amounts of food in an attempt to contradict their diagnosis as a “food addict.” However, while such factors may have played a role, findings from study 2 suggest that the effect of the feedback on food intake was primarily driven by increased dietary concern.

Exposure to a food addiction explanation of obesity has recently been shown to reduce weight stigma and blame towards individuals who have obesity 35. This suggests that there may be beneficial effects of believing in food addiction. To our knowledge, this study is the first to provide insight into the causal influence of personal food addiction beliefs on eating behavior. Notably, we tested a nonclinical sample of female participants, and it is now important to apply this approach to males and populations who have obesity. Indeed, our belief manipulation may have an opposite effect on calorie intake in individuals who have obesity, particularly if the feedback is congruent with pre‐existing personal beliefs about eating behavior. Future research should also consider the longevity of the effect. Notably, in study 2 we did not uncover any significant effect of personal food addiction beliefs on longer‐term dieting intentions. Previous research has shown that attempts to restrict food intake over longer time periods can be futile by exacerbating cravings and promoting disinhibited eating patterns 11, 36, 37. On this basis, believing oneself to be a food addict might not be conducive to successful longer‐term dietary control and weight management.

In conclusion, we found that believing oneself to be a food addict was associated with a subsequent reduction in calorie intake. By causing individuals to become more concerned about their eating behavior, personal food addiction beliefs may help minimize the extent to which people expose themselves to food. Further research should establish the longer‐term effects of personal food addiction beliefs and the potential implications for dietary control and obesity.

## Supporting information

Supporting Information

## Footnotes

1 The analyses were rerun to include the two participants who did not believe in food addiction and guessed the aims of the study, and the results did not change.

2 Results are reported as mean ± standard deviation.

3 Analysis using continuous YFAS sum scores (see Ref. 29) also revealed no significant difference between conditions, *t*_(59)_ = 1.11, *P* = 0.270.

4 All findings remained the same when the five participants who guessed the aims of the study, or who did not believe in the concept of food addiction, were included in the analyses.

5 Participants also did not differ between conditions when YFAS data were analyzed using continuous sum scores, *F*_(2,82)_ = 0.961, *P* = 0.387.
