# Supplementary material for: Believing in food addiction: Helpful or counterproductive for eating behavior?
Source: Obesity (Silver Spring). 2016 May 5;24(6):1238–43. doi: 10.1002/oby.21499 (PMC5084740; doi:10.1002/oby.21499)
Supplement: Supplementary file 1 — Supporting Information [file OBY-24-1238-s001.docx]

**Supporting Information**

**Mood and Taste ratings**…………………………………………………………………. 1

**Mediation analysis procedure**……………………………………….…………….……..2

Table S1. (indirect effects of condition on calorie intake)…………….…...………………3

**Supplemental analyses**………..…………..........................................................................4

**Study 1** ………………………………..…………...................................................4

**Study 2**…………………………………………..………………………….……..4-5

**Additional references**..........................................................................................................5

**Mood and Taste ratings**

Participants provided ratings, using 100mm VAS scales, for the following states:

I feel tense/anxious/nervous/on edge

I feel mentally alert/attentive/observant

I feel motivated

I feel hot/sweaty

I feel physically tired

I feel clearheaded

I feel miserable/dejected

I feel stressed

I feel friendly/sociable

I feel mentally fatigued/drained/worn out

I feel relaxed/calm/at ease

I feel strange/weird/not my usual self

I feel sleepy/drowsy/half awake

I feel energetic/active/lively

My head aches/I feel head-achey

I feel able to take on a physically demanding task

I feel able to concentrate/able to focus

I feel angry/cross/annoyed

I feel lethargic/sluggish

I feel cheerful/happy

My heart is pounding/racing

Participants completed the following taste ratings, for the chocolate and crisps, using 100mm VAS scales:

Sweet

Salty

Enjoyable

Fresh

Bland

Crunchy

Crumbly

**Mediation analyses procedure**

The following procedure was used to examine the effect of each condition on calorie intake and the mediating roles of dietary concern and time taken to complete the taste task. Firstly, to explore the indirect effects of the low-addiction condition, this condition was dummy coded as 1, while average- and high-addiction conditions were dummy coded as 0. Secondly, to explore the indirect effects of the high-addiction condition, this was dummy coded as 1, while average- and low- addiction conditions were coded as 0. The two proposed mediators (i.e. dietary concern and time-taken) and the dependent variable (i.e. calorie intake) were then log-transformed prior to analysis. This was to ensure that the data met parametric assumptions, and for easier comparison of effects.

The model was then computed using PROCESS (model 6) (28) by entering between condition comparisons (i.e. low vs average; high vs average) as independent variables, and calorie intake as the dependent variable. As dietary concern was expected to influence calorie intake via its effect on time taken, dietary concern and time taken were entered as the first and second mediators, respectively. The model was conducted twice to provide coefficients for each between-condition comparison. As recommended by Hayes and Preacher (29), when exploring the indirect effects of the high-addiction condition, the low addiction condition was entered as a covariate in the mediation model, and vice versa.

Due to the asymmetric distribution associated with mediation analyses, the total indirect effect was tested using a nonparametric bootstrapping procedure (*n*=10,000 samples). This procedure establishes 95% confidence intervals which can be used to deduce levels of significance. Specifically, the total indirect effect of the model may be interpreted as significant (*p*<.05) if the resulting CIs do not contain zero (1) (Table S1).

Table S1. *Indirect effects of condition on calorie intake via dietary concern and time taken to complete the taste task.*

| Indirect effect | Comparison | *B* (SE) | CL.95 (lower bound) | CL.95 (upper bound) |
| --- | --- | --- | --- | --- |
| Total indirect effect | Low vs. Average | -0.02(.07) | -0.16 | 0.12 |
|  | High vs. Average | -.21(.08)* | -0.36 | -0.07 |
| Indirect effect 1 | Low vs. Average | -.02(.03) | -0.08 | 0.02 |
|  | High vs. Average | .02(.03) | -0.03 | 0.10 |
| Indirect effect 2 | Low vs. Average | -0.05(.07) | -0.19 | 0.08 |
|  | High vs. Average | -.17(.08)* | -0.33 | -0.02 |
| Indirect effect 3 | Low vs. Average | .04(.03) | 0.00 | 0.13 |
|  | High vs. Average | -.06(.03)* | -0.13 | -0.01 |

*Significant effects at p<.05

Indirect effect 1: Condition -> dietary concern -> intake
Indirect effect 2: Condition -> time taken -> intake

Indirect effect 3: Condition -> dietary concern -> Time taken -> intake

**Supplemental analyses**

**Study 1.**

***Food ratings.*** Group differences in liking, craving, desire to eat, difficulty to resist, and post-consumption enjoyment ratings were explored using 2 x 2 mixed design ANOVAs. For each analysis, food (*i.e.,* crisps and chocolate) was entered as the within subjects variable, and condition (*i.e.,* high- and low-addiction) was entered as the between subjects variable. There was no main effect of condition (*ps* > .250), and no condition x food interactions (*ps* >.215), on any rating. There was a main effect of food such that chocolate was rated higher on all appetitive scales than crisps (*ps* < .034).

***Hunger and fullness.*** Hunger and fullness ratings were analysed using 2 x 2 mixed design factorial ANOVAs. Time (*i.e.,* before and after the taste task) was the within subjects variable, and condition (*i.e.,* high- and low-addiction) was the between subjects variable. There was no main effect of condition (*ps*>.250), and no condition x time interaction (*ps* >.229), on hunger and fullness ratings. There was a main effect of time on hunger, *F*(1,60)=56.36, *p*<.001, $\eta_{p}^{2}$=.48, and fullness, *F*(1,60)=35.79, *p*<.001, $\eta_{p}^{2}$=0.37. Specifically, prior to the taste task, hunger ratings were significantly greater (M=61.69, SD=38.35 mm), and fullness ratings significantly lower (M=24.79 ± 20.12 mm), than after the taste task (Hunger: M=38.35 ± 24.68 mm; Fullness: M=44.54 ± 25.84 mm).

***Mood and taste.*** Multivariate ANOVAs revealed no main effect of condition on mood, *F*(21,40) =1.00, *p*>.250, $\eta_{p}^{2}$=.345, or taste ratings, *F*(14, 47) = .622, *p*>.250, $\eta_{p}^{2}$=.156.

**Study 2.**

***Food ratings.*** A 3 x 2 mixed-design ANOVA revealed no main effect of condition (*ps*>.086), and no condition x food interactions (*ps*>.250), on liking, craving, desire to eat, difficulty to resist, or post-consumption enjoyment ratings. There was a main effect of food such that chocolate was rated higher than crisps on all scales (*ps* <.001) except for ‘difficulty to resist’ (*F*(2,82)=0.48, *p*=.092).

***Hunger and fullness.*** Mixed-design ANOVAs revealed no main effect of condition on hunger or fullness ratings (*ps*>.250). There was a main effect of time such that hunger ratings decreased, *F*(1,82)=65.94, *p*<.001, $\eta_{p}^{2}$=.446, and fullness ratings increased, *F*(1,87)=65.93, *p*<.001, $\eta_{p}^{2}$=.446, following the taste task. A condition x time interaction was observed for hunger ratings, *F*(2,82)=5.31, *p*=.007, $\eta_{p}^{2}$=.12. To examine this further, changes in hunger ratings, before and after the taste task, were calculated for each participant. A univariate ANOVA revealed a main effect of condition on hunger change, *F*(2,82)=5.31, *p*=.007, $\eta_{p}^{2}$ =.115. Pairwise comparisons revealed that hunger ratings declined significantly less, following the taste task, in the high-addiction condition (M=9.39 ± 19.34 mm), compared to the low- (M=25.24 ± 29.78 mm), *p*=.016, *d*=.63, and average-addiction (M=29.32 ± 22.03 mm), *p*=.003, *d*=.96, conditions. Hunger change did not differ between average- and low- addiction conditions, *p*=.526, *d*=.16.

There was no time x condition interaction on fullness ratings, *F*(2,82)=2.56, *p*=.083, $\eta_{p}^{2}$ =.059.

***Mood and taste.*** Finally, multivariate ANOVAs revealed no main effect of condition on mood, *F*(42,124)=1.05, *p*>.250, $\eta_{p}^{2}$=.262, or taste ratings, *F*(28,138)=0.66, *p*>.250, $\eta_{p}^{2}$=.118.

***Self-control and Dieting Intentions****.* Separate univariate ANOVAs revealed no main effect of condition on self-control ratings, *F*(2,82)=1.90, *p*=.158, η_p_² = .04 nor on the dieting intention scale (DIS) scores, *F*(2,82)=0.99, *p*=.377, η_p_²=.02. Thus, the effect of experimental condition on calorie consumption does not appear to be caused by changes in self-control ratings or future dieting intentions.

**Note**

The raw data files associated with these studies may be viewed at: [10.17638/datacat.liverpool.ac.uk/99](http://dx.doi.org/10.17638/datacat.liverpool.ac.uk/99)

**Additional references**

1. MacKinnon DP, Lockwood CM, Hoffman JM, West SG, Sheets V. A comparison of methods to test mediation and other intervening variable effects. *Psychol Methods* 2002; 7: 83–104.
